# Integrated transcriptomic and functional analyses identify LAIR1 as a key immunoregulatory molecule in postoperative sepsis

**DOI:** 10.3389/fimmu.2026.1931628

**Published:** 2026-10-14

**Authors:** Tao Liu, Yang Wang, Liyun Zeng, Xuemei Miao, Yu Peng, Jingying Yu, Jiaying Li, Qian Zhao, Ziyan Chen, Ruping Dai, Hui Li

**Affiliations:** 1 Department of Anesthesiology, The Second Xiangya Hospital, Central South University, Changsha, Hunan, China; 2 Anesthesiology Research Institute, Central South University, Changsha, Hunan, China

**Keywords:** bioinformatics, immune dysregulation, LAIR1, monocytes, postoperative sepsis

## Abstract

**Background:**

Postoperative sepsis arises from a dysregulated host response to infection following surgical stress. Current non-specific biomarkers offer insufficient predictive value, highlighting the need to identify molecular signatures that may facilitate earlier recognition of postoperative sepsis while providing insight into the underlying immune regulatory mechanisms.

**Methods:**

Transcriptomic datasets comprising postoperative septic and non-septic surgical cohorts, together with independent cohorts of sepsis and non-infectious systemic inflammation, were analyzed using Weighted Gene Co-expression Network Analysis and machine-learning approaches to identify hub genes. A diagnostic nomogram was constructed and validated across independent cohorts. A murine postoperative endotoxemia model (tibial fracture surgery followed by LPS challenge) was used for *in vivo* validation. Single-cell RNA sequencing analysis and LAIR1 antibody blockade were performed to assess cellular and functional relevance.

**Results:**

A four-gene signature comprising LAIR1, PLAC8, ACSS2, and PCOLCE2 was identified. The resulting model achieved AUCs of 0.865 in GSE131761, 0.965 in GSE74224, 0.827 in E-MTAB-1548, 0.929 in GSE65682, and 0.997 in GSE77167. Among the four genes, LAIR1 showed the most sustained upregulation in peripheral blood and multiple organs during postoperative endotoxemia. Single-cell analysis identified monocytes as the predominant circulating source of LAIR1. LAIR1 blockade increased systemic cytokine release, aggravated multiorgan injury, and reduced survival.

**Conclusions:**

The four-gene signature reproducibly distinguished sepsis from infection-negative systemic inflammation across surgical and broader ICU cohorts. LAIR1 exhibited monocyte-enriched expression and a protective immunoregulatory role in postoperative endotoxemia, supporting its relevance to immune dysregulation of sepsis.

## 1 Introduction

Sepsis is a life-threatening organ dysfunction caused by a dysregulated host response to infection, accounting for approximately 19.7% of global mortality (1, 2). In surgical patients, postoperative sepsis occurs in approximately 3.2% of cases and remains a major contributor to intensive care unit mortality (3–6). Unlike community-acquired sepsis, postoperative sepsis develops in the context of surgery-induced immune activation, in which sterile inflammatory responses and subsequent infectious insults interact to drive complex immune dysregulation (7, 8). Surgical injury induces a preconditioned immune state characterized by profound systemic immune activation prior to infection, thereby reshaping subsequent host inflammatory responses and contributing to heterogeneous disease trajectories (9). However, the regulatory programs governing postoperative immune remodeling remain poorly defined.

Current biomarkers, including procalcitonin (PCT), C-reactive protein (CRP), and interleukin-6 (IL-6), primarily reflect nonspecific inflammatory responses and provide limited insight into the immune regulatory mechanisms underlying postoperative sepsis (10–12). Although transcriptomic approaches have improved molecular classification of sepsis, most studies remain limited to static disease states and fail to capture dynamic immune remodeling from surgery-induced inflammation to infection-associated dysregulation (13, 14). Consequently, conserved immune programs associated with postoperative sepsis, as well as robust molecular frameworks for risk stratification and immune regulation, remain insufficiently defined.

Within this context, innate immune responses undergo profound functional remodeling, integrating inflammatory amplification with subsequent immunoregulatory adaptation (15–17). In particular, monocyte-centered programs play a central role in coordinating systemic immune responses under inflammatory stress, yet the molecular circuits governing their adaptation in surgery-conditioned states remain incompletely defined. Among inhibitory immune pathways, leukocyte-associated immunoglobulin-like receptor 1 (LAIR1/CD305), an ITIM-containing receptor broadly expressed in myeloid cells, has emerged as a potential regulator of immune suppression through engagement with collagen and complement components such as C1q (18). However, whether LAIR1 functions within monocyte-centered regulatory programs to control immune adaptation during postoperative immune reprogramming remains unclear.

In this study, we performed an integrative transcriptomic analysis combining WGCNA and machine-learning approaches to identify LAIR1, PLAC8, ACSS2, and PCOLCE2 as a conserved gene set associated with postoperative sepsis. A four-gene nomogram-based model was constructed and validated across independent cohorts. Using a postoperative endotoxemia model and single-cell RNA sequencing analysis, we demonstrate that LAIR1 is selectively enriched within monocyte-associated innate immune compartments and dynamically linked to inflammatory regulation during disease progression. Functional blockade experiments further show that disruption of LAIR1 exacerbates systemic inflammation and multiorgan injury. Collectively, these findings identify LAIR1 as a monocyte-associated immunoregulatory molecule that contributes to surgery-induced immune dysregulation and regulates postoperative inflammatory responses.

## 2 Materials and methods

### 2.1 Dataset search and eligibility criteria

Publicly available human transcriptomic datasets were systematically searched in the Gene Expression Omnibus (GEO, https://www.ncbi.nlm.nih.gov/geo/) and ArrayExpress/BioStudies databases (https://www.ebi.ac.uk/arrayexpress/). Because datasets specifically designed for postoperative sepsis are limited, a tiered search strategy was used. The initial GEO search using (“postoperative” OR “postsurgical” OR “post-surgical”) AND (“sepsis” OR “critically ill”) AND “Homo sapiens”[porgn:_txid9606], returned 10 records. GSE131761 and GSE74224 met the prespecified eligibility criteria. ArrayExpress/BioStudies was searched using (“postoperative” OR “postsurgical” OR “post-surgical”) AND (“sepsis” OR “critically ill”) AND (“Homo sapiens”), yielding 3 candidate records, from which E-MTAB-1548 was retained. The search was subsequently expanded to postoperative infection using (“postoperative” OR “postsurgical” OR “post-surgical”) AND (“infection”) AND “Homo sapiens”[porgn:_txid9606], which identified GSE77167. A broader search for human blood sepsis transcriptomic datasets using (“sepsis” OR “critically ill”) AND (“whole blood” OR “peripheral blood”) AND (“gene expression” OR “transcriptome”) AND “Homo sapiens”[porgn:_txid9606] AND gse[Entry Type], which identified GSE65682. Candidate datasets were screened by reviewing study design, sample source, case and comparator definitions, sampling time, sample-level metadata, and the corresponding original publications. The final search update was conducted on September 29, 2026.

Datasets were eligible if they contained (i) human clinical bulk transcriptomic data derived from whole blood, peripheral blood leukocytes, or peripheral blood mononuclear cells (PBMCs); (ii) a clinically defined infectious group, including postoperative infection, sepsis, or septic shock, together with a clinically relevant comparator group; (iii) available clinical metadata, where available, including sample timing relative to surgery, ICU admission, or infection/sepsis diagnosis; and (iv) publicly accessible expression data with adequate phenotype annotation. For datasets containing multiple clinical populations, only the prespecified eligible sample-level subsets were included.

Datasets were excluded if they involved animal or *in vitro* experiments, non-blood tissues, exclusively preoperative samples, lacked either an eligible infectious group or a clinically relevant comparator, or did not provide sufficient expression or phenotype information. Studies with healthy controls only were not used for the primary postoperative comparisons.

### 2.2 Cohort selection and study design

Five datasets were included and assigned distinct roles according to their clinical relevance to postoperative sepsis. GSE131761 (13) served as the primary postoperative discovery and model-development cohort and was used for weighted gene co-expression network analysis (WGCNA), candidate-gene identification, and nomogram development. After excluding 15 healthy-control samples, 81 postoperative septic-shock samples and 33 postoperative non-septic-shock samples were included. According to the original study, septic shock was defined using the Sepsis-3 criteria (1), and a positive microbiological culture was an additional inclusion requirement for the septic-shock group.

GSE74224 (14) was used as a secondary discovery cohort and included 74 critically ill patients with sepsis and 31 postsurgical patients with infection-negative systemic inflammation. Final diagnoses were retrospectively adjudicated by intensive care physicians using available clinical and microbiological information in accordance with the 1992 ACCP/SCCM consensus criteria.

E-MTAB-1548 (21) was selected as the principal external validation cohort because of its independent surgical population and postoperative clinical setting. After exclusion of 15 healthy controls, 82 patients with postoperative sepsis and 58 patients with postoperative non-infectious systemic inflammation were included. Clinical classifications followed the original study and the 1992 ACCP/SCCM consensus criteria, with sepsis representing infection-associated systemic inflammation and the comparator group representing systemic inflammatory response syndrome (SIRS) without evidence of infection.

GSE65682 (19), a large and clinically heterogeneous whole-blood transcriptomic cohort from the Molecular Diagnosis and Risk Stratification of Sepsis (MARS) study, was included as a complementary external validation cohort. Because of the heterogeneity of the overall dataset, only the predefined abdominal_sepsis_and_controls subset was analyzed, comprising 51 patients with abdominal sepsis (abdo_s) and 42 postoperative gastrointestinal controls (ctrl_GI). This subset provided a clinically relevant abdominal/gastrointestinal comparison and was used to assess the reproducibility of the candidate-gene signature in distinguishing abdominal sepsis from non-infectious postoperative inflammation.

GSE77167 (20) was included as an additional external validation cohort because postoperative infection and postoperative sepsis share infection-associated host immune responses occurring in the setting of surgery-induced inflammation. Based on the sample-level clinical annotations provided in GEO, the analysis included 32 patients who developed intra-abdominal infection after colorectal cancer surgery and 30 patients with an uncomplicated postoperative course. The infection group comprised patients with anastomotic leakage or intra-abdominal abscess. This dataset was used to evaluate the ability of the four-gene signature to discriminate postoperative infection from postoperative sterile inflammation.

Detailed cohort characteristics, sampling information, sample-selection rules, and analytical roles are summarized in **Supplementary Tables 1** and **2**.

### 2.3 Transcriptomic data preprocessing

Expression data from each cohort were processed independently. Preprocessed expression matrices provided by the original repositories were used, and log2 transformation was applied only when required. Probe identifiers were mapped to gene symbols according to the corresponding platform annotations. Probes without valid gene-symbol annotations were removed, and multiple probes mapping to the same gene were summarized using the median expression value. No cross-cohort batch correction was performed because each external dataset was analyzed independently.

### 2.4 Animals

Wild-type male C57BL/6J mice (8–10 weeks old) were obtained from Changsha SLA Laboratory Animal Co., Ltd. Animals were housed under specific pathogen-free (SPF) conditions at 22–25 °C with a 12 h light/dark cycle, with 4–6 mice per cage and ad libitum access to food and water. Mice were randomly assigned to experimental groups as described below. All experimental procedures were approved by the Institutional Animal Care and Use Committee (IACUC) of the Second Xiangya Hospital (No. 20260798), Central South University, and were conducted in accordance with the NIH Guide for the Care and Use of Laboratory Animals.

### 2.5 Identification of differentially expressed genes and enrichment analysis

DEGs between the postoperative sepsis and postoperative non-sepsis groups were identified using the “limma” package. The significance thresholds were set at a p value < 0.05 and log_2_ fold change (|log2 FC|) > 0.5. Volcano plots were generated using “ggplot2”, Functional enrichment; including Gene Ontology (GO) and Kyoto Encyclopedia of Genes and Genomes (KEGG) pathways, was conducted using Metascape (http://metascape.org).

### 2.6 Weighted gene co-expression network analysis

Co-expression networks were constructed using the “WGCNA” R package, based on the GSE131761 dataset. After outlier removal via sample clustering, a soft thresholding power of *β* = 14 was selected to ensure a scale-free topology. Modules were identified using the dynamic tree-cut method with a minimum module size of 30 and a merge threshold of 0.25. The association between modules and clinical traits (postoperative sepsis vs. postoperative non-sepsis) was evaluated using Pearson correlation analysis. Genes within the most significant modules were extracted for identification of downstream hub genes. Additionally, the module sensitivity was set to 1.0, the module pooling threshold was set to 0.5, and the minimum module gene number set to 30. Finally, 9 co-expression modules were identified.

### 2.7 Machine learning-based hub gene identification

Different machine learning algorithms were applied to identify hub genes. First, candidate genes were selected by intersecting DEGs with genes from the hub modules identified using WGCNA. The hub genes were further refined using three methods: SVM-RFE with the “e1071” package (22), the random forest (RF) algorithm using the “randomForest” package (23), and the LASSO algorithm with the “glmnet” package (24). The genes overlapping across these three models were identified as the final hub genes for further analysis. Genes consistently identified by all three models were defined as final hub genes.

### 2.8 Nomogram construction and validation

A diagnostic model incorporating the four candidate genes—LAIR1, PLAC8, ACSS2, and PCOLCE2—was developed using data from GSE131761, which served as the model-development cohort. The model was visualized as a nomogram using the “rms” R package (25). Model performance was quantified using the C-index with 1000 bootstrap resamples for internal validation. Calibration curves were generated to assess the agreement between predicted and observed probabilities. Decision Curve Analysis (DCA) and Clinical Impact Curves (CIC) were performed using the “rmda” package to evaluate the clinical net benefit and utility (26, 27).

### 2.9 ROC curve analysis

A receiver operating characteristic (ROC) curve is a plot in which the sensitivity (true positive rate) is plotted as a function of 100% minus specificity (false positive rate) at different cutoff points of a specific parameter. In the present study, we used the “pROC” package (28) to generate ROC curves; the diagnostic capacities of the hub genes and nomogram model were examined using the area under the curve (AUC).

### 2.10 Single-cell RNA-seq and intercellular communication analysis

Human PBMC single-cell RNA-sequencing data were obtained from the publicly available GSE167363 dataset. The dataset comprised 12 PBMC samples from seven individuals, including two healthy controls and five patients with gram-negative bacterial sepsis. The sepsis cohort included three survivors and two non-survivors, each sampled at sepsis recognition (T0) and 6 h after recognition (T6), resulting in ten longitudinal sepsis samples. The feature-barcode matrices of all 12 samples were imported into Seurat (version 4.0) to construct a combined Seurat object. The percentage of mitochondrial transcripts in each cell was calculated using the PercentageFeatureSet function with the pattern ^MT-. For quality control, cells were retained if they had more than 500 but fewer than 5,000 detected genes, fewer than 25,000 total UMI counts, and less than 15% mitochondrial transcripts (nFeature_RNA > 500 & nFeature_RNA < 5000 & nCount_RNA < 25000 & percent.mt < 15). After quality-control filtering, the final Seurat object contained 53,934 cells and 20,696 detected genes for downstream analysis.

Gene-expression data were normalized using the LogNormalize method, followed by identification of highly variable features, data scaling, and principal component analysis (PCA). To reduce technical variation among samples, batch effects were corrected using the Harmony algorithm, with sample identity specified as the batch variable. The Harmony-corrected embeddings were subsequently used for nearest-neighbor graph construction, unsupervised clustering, and uniform manifold approximation and projection (UMAP) visualization. Cell clusters were identified using unsupervised clustering and annotated based on canonical marker genes. The relative proportions of each cell type across experimental samples were calculated and visualized as stacked bar plots. The expression profiles of target genes were displayed using FeaturePlot and VlnPlot. For group-wise comparisons within specific cell lineages, monocytes were subsetted, and differential expression analysis was conducted using the Wilcoxon rank-sum test.

Cell–cell communication analysis was conducted using “CellChat (v1.1.0)” with the CellChatDB.human database as a reference. Sepsis survivors and non-survivors were combined into a single sepsis group. Communication probabilities were inferred by integrating gene expression data, and the overall signaling strength, including incoming and outgoing interactions, was quantified and compared between Healthy Control and Sepsis groups. Differential signaling pathways and monocyte-associated interaction networks were identified and visualized using circular plots, two-dimensional scatter plots, and heatmaps.

To account for data sparsity inherent to single-cell RNA sequencing, monocytes were ranked according to LAIR1 expression levels and stratified into 50 bins of equal size, as previously described (29, 30). Consistent results were observed using alternative binning strategies (e.g., 30 or 80 bins). The average expression levels of LAIR1 and selected immunoregulatory genes (e.g., IL10, CD163) were calculated within each bin, and correlation analyses were performed using these averaged values.

### 2.11 Surgical procedures and induction of postoperative endotoxemia model

Postoperative endotoxemia model was established by combining surgical trauma with a subsequent endotoxemic challenge, following previously described protocols (31). Briefly, mice were anesthetized with 1% pentobarbital (100 mg/kg, i.p.) and received buprenorphine analgesia (0.1 mg/kg, s.c.). A longitudinal incision was made along the lateral aspect of the tibia. An intramedullary stainless steel pin (0.38 mm in diameter) was inserted into the medullary canal, followed by osteotomy at the mid-distal third of the tibia to induce tibial fracture with internal fixation. Twenty-four hours after surgery, mice were intraperitoneally injected with LPS (E. coli LPS O55:B5; Sigma-Aldrich, L2880, 15 mg/kg) to induce acute endotoxemia. Control mice received an equal volume of PBS, and subcutaneous saline (0.5 mL) was administered for fluid resuscitation after LPS injection.

Peripheral blood and tissue samples (lung, liver, and kidney) were collected at 6 h and 24 h after LPS administration for gene expression and histological analyses. Separate cohorts were used for survival and mechanistic analyses. Mice in survival experiments were not subjected to tissue collection, whereas independent cohorts were sacrificed at predefined time points (6 h and 24 h post-LPS) for molecular and histological assessments.

### 2.12 Histological examination and lung injury score

The lungs, liver, and kidneys were collected at 6 h and 24 h after LPS challenge, then fixed in 4% paraformaldehyde, embedded in paraffin, and sectioned at 4 μm. Hematoxylin and eosin (H&E) staining was performed for morphological assessments. Histological lung injury scores were determined based on alveolar hyperemia, alveolar edema, neutrophil infiltration, and alveolar septum thickening (32). Histological liver injury was determined based on the degree of liver cell cytoplasmic vacuolation, hepatic sinus congestion, and liver cell necrosis (33). Histological kidney injury was assessed based on the degree of damaged tubules and the atrophic glomerulus score (34).

### 2.13 Immunofluorescence and immunohistochemical analyses

For immunofluorescence staining, lung sections were subjected to antigen retrieval and permeabilized and blocked with 5% BSA containing 0.2% Triton X-100. The sections were then incubated overnight at 4 °C with primary antibodies against LAIR1 (1:400; Proteintech, 13297-1-AP) and F4/80 (1:500; Abcam, ab90247). The colocalization of LAIR1 and F4/80 was evaluated to assess LAIR1 expression in pulmonary macrophages. Images were acquired under identical settings and analyzed using Fiji. LAIR1–F4/80 colocalization was quantified using thresholded Manders’ M1, with LAIR1 designated as channel 1 and F4/80 as channel 2. Multiple fields were averaged for each mouse.

For IHC, lung, liver, and kidney sections underwent antigen retrieval, followed by quenching of endogenous peroxidase activity with 3% H_2_O_2_. After blocking with 5% BSA/0.2% Triton X-100, the sections were incubated with anti-LAIR1 primary antibody (1:400, Proteintech, 13297-1-AP) overnight at 4 °C, followed by incubation with biotinylated secondary antibody (1:200, Thermo Fisher, 31820) and ABC reagent (Vector, PK-6101). The signal was developed using DAB.

### 2.14 RNA extraction and RT-qPCR

Total RNA was extracted from peripheral blood mononuclear cells (PBMCs) and various tissues using the TRIzol reagent (Thermo Fisher Scientific, 15596018CN). cDNA was synthesized using a RevertAid First Strand cDNA Synthesis Kit (Thermo Fisher Scientific, K1622). Quantitative PCR was performed on a CFX96 Touch system (Bio-Rad) using SYBR Green Master Mix (Novoprotein, E301). Relative gene expression was calculated using the 2^-ΔΔCT^ method and was normalized to that of GAPDH. The primers used are listed in **Supplementary Table 3**. To visualize the spatio-temporal expression patterns of hub genes across multiple organs and time points, qPCR data were presented as a heatmap. The mean relative expression values (fold change) for each target gene—*Lair1*, *Plac8*, *Acss2*, and *Pcolce2*—were calculated relative to the Sham group at the corresponding time points. These fold-change values were then log_2_ transformed to ensure a symmetrical representation of upregulated and downregulated genes. The heatmap was generated using GraphPad Prism (version 8.0).

### 2.15 ELISA

Serum levels of IL-6, IL-1β, and TNF-α were quantified using ELISA kits (Proteintech: KE10007, KE10003, and KE10002) according to the manufacturer’s protocols.

### 2.16 Isolation of peripheral blood mononuclear cells

PBMCs were isolated from mouse peripheral blood collected at 6 h and 24 h after LPS injection via retro-orbital bleeding under deep anesthesia into anticoagulant-coated tubes. PBMCs were obtained by density gradient centrifugation using PBMC separation medium (Solarbio, P6340). Briefly, whole blood was diluted 1:1 with phosphate-buffered saline (PBS) and carefully layered over the separation medium. Samples were centrifuged at 1000 × g for 30 min at room temperature. The mononuclear cell layer at the interface was collected and washed twice with PBS (250 × g, 5 min). The isolated cells were lysed in TRIzol reagent (Thermo Fisher Scientific, 15596018CN), and total RNA was extracted according to the manufacturer’s instructions.

### 2.17 Neutralizing antibody intervention *in vivo*

Mice were randomly assigned to receive either an anti-LAIR1 antibody (R&D Systems, MAB100921) or an isotype control IgG (R&D Systems, AB-105-C) via intraperitoneal (i.p.) injection at a dose of 200 μg per mouse. The dosing regimen was based on a previously published study demonstrating effective *in vivo* blockade of LAIR1 signaling using antibody-mediated inhibition (35). The antibody was administered according to a multi-dose regimen. The first dose was given 1 h before tibial fracture surgery, and the second dose was administered 1 h prior to LPS injection (postoperative day 1, POD 1). Subsequent doses were delivered once daily until the experimental endpoint.

For survival analysis, mice received daily antibody injections for 5 consecutive days starting on the day of fracture surgery. For molecular and histological analyses, mice were treated for 3 consecutive days, and serum as well as liver, lung, and kidney tissues were collected 1 h after the final injection on day 3.

### 2.18 Western blotting

Total proteins were extracted from lung tissues, and protein concentrations were determined using a BCA Protein Assay Kit (Beyotime, P0012) according to the manufacturer’s instructions. Equal amounts of protein were separated by 10% SDS-polyacrylamide gel for electrophoresis and subsequently transferred onto polyvinylidene difluoride membranes. The membranes were incubated with primary antibodies against LAIR1 (1:1000; Proteintech, 85010-1-RR), PLAC8 (1:1000; Proteintech, 12284-1-AP), ACSS2 (1:5000; Proteintech, 16087-1-AP), PCOLCE2 (1:1000; CUSABIO, CSB-PA017632LA01HU), and GAPDH (1:1,000; Abcam, ab8245). After incubation with the appropriate horseradish peroxidase-conjugated secondary antibodies, immunoreactive bands were visualized using a chemiluminescence imaging system (CLINX, Shanghai, China).

### 2.19 Statistical analysis

All bioinformatic analyses were performed in R software (v4.2.3). Differential expression analysis was conducted using the limma package. Student’s t test was used for two-group comparisons of candidate gene expression where applicable, and correlations were evaluated using Spearman’s rank correlation. ROC curve analysis was conducted using the “*pROC”* package to calculate AUCs with 95% confidence intervals. A diagnostic nomogram was constructed using the “rms*”* package and validated in independent cohorts.

Experimental data from animal studies are presented as mean ± SEM and were analyzed using GraphPad Prism (version 8.0). Differences between two groups were assessed using unpaired two-tailed Student’s t-test. Comparisons among multiple groups were performed using one-way ANOVA followed by Tukey’s *post-hoc* test. Experiments involving treatment and time as two independent factors were analyzed using ordinary two-way ANOVA, including the treatment × time interaction, followed by Tukey’s multiple-comparisons test. Survival analysis was conducted using the Kaplan–Meier method with log-rank (Mantel–Cox) test. A p value < 0.05 was considered statistically significant.

## 3 Results

### 3.1 Cross-cohort transcriptomic analysis and machine-learning prioritization of candidate genes associated with postoperative sepsis

To identify candidate genes associated with postoperative sepsis, we first performed differential expression analysis in GSE131761, which compared patients with postoperative septic shock with those with postoperative non-septic shock. A total of 443 DEGs were identified, including 216 upregulated and 227 downregulated genes (**Figure 1A**). We subsequently analyzed GSE74224, as a complementary discovery cohort, comprising critically ill patients with confirmed sepsis and patients with postoperative infection-negative systemic inflammation. Differential expression analysis identified 418 DEGs, including 340 upregulated and 78 downregulated genes (**Figure 1B**). Although one cohort comprised patients with postoperative sepsis and the other included patients with sepsis of diverse etiologies, both comparisons evaluated sepsis-associated transcriptional alterations against non-infectious postoperative inflammation. We therefore intersected their DEG sets to prioritize signals reproducible across cohorts. This analysis yielded 75 shared DEGs (**Figure 1C**), which were taken forward for further investigation in the primary postsurgical cohort. These shared genes were predominantly enriched in innate immune programs, particularly neutrophil activation, bacterial response pathways, and metabolic regulation (**Supplementary Figures 1A, B**). Network analysis of enriched terms further supported a dominant myeloid inflammatory signature (**Supplementary Figure 1C**).

**Figure 1:**
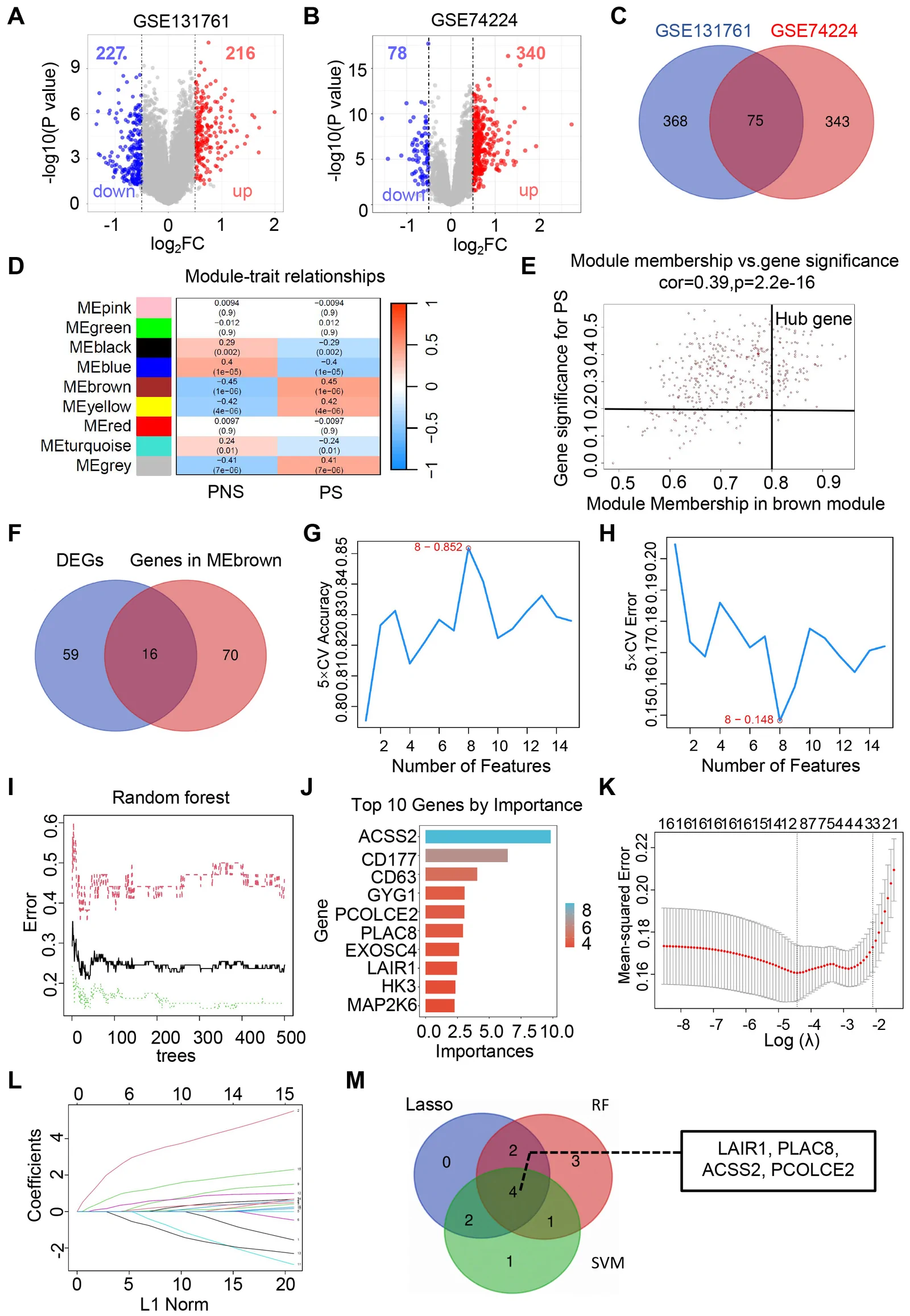
Cross-cohort transcriptomic analysis and machine-learning prioritization of candidate genes associated with postoperative sepsis. **(A, B)** Volcano plots displaying DEGs in the GSE131761 **(A)** and GSE74224 **(B)** datasets. **(C)** Venn diagram of DEGs in GSE131761 and GSE74224. **(D)** Heatmap of module–trait relationships in WGCNA. Each cell contains the correlation coefficient (upper) and the corresponding p-value (lower) between modules and clinical phenotypes (PNS and PS). **(E)** Scatter plot illustrating the correlation between module membership (MM) and gene significance (GS) in the brown module. **(F)** Venn diagram showing the overlap (16 genes) between the 75 common DEGs and genes within the hub gene of brown module. **(G, H)** Eight gene signatures were identified by SVM-RFE analysis with an accuracy of 0.852 **(G)**, Error of 0.148 **(H)**. **(I, J)** Random Forest (RF) analysis. The error rate plot **(I)** shows the stability of the model across 500 trees. The variable importance plot **(J)** ranks the top 10 genes based on their Mean Decrease Gini/Accuracy scores. **(K)** Cross-validation to select the optimal tuning parameter log (Lambda) in LASSO regression analysis. **(L)** LASSO coefficient profiles of candidate genes. **(M)** Venn diagram of four hub genes shared by the SVM-RFE, RF, and LASSO algorithms. DEGs differentially expressed genes. PNS postoperative non-sepsis, PS postoperative sepsis.

To identify co-expression modules specifically associated with postoperative sepsis, WGCNA was subsequently performed in GSE131761 after the removal of outlier samples. Using a soft-thresholding power of β = 14, nine co-expression modules were identified (**Supplementary Figures 2A–C**). Among these modules, the MEBrown module showed the strongest association with postoperative sepsis (r = 0.45, p = 1×10^-6^) and was enriched in neutrophil degranulation, metabolic regulation, and platelet activation pathways (**Figure 1D**; **Supplementary Figures 2D, E**). Application of GS and MM thresholds (|GS| > 0.20, |MM| > 0.80) identified 86 high-confidence hub genes (**Figure 1E**). Intersection with the 75 consensus DEGs yielded 16 core candidate genes (**Figure 1F**).

To further prioritize candidate features, three complementary machine-learning feature-selection methods—SVM-RFE, random forest, and LASSO regression—were applied to the 16 candidate genes. These models independently selected 8, 10, and 8 candidate genes with stable predictive performance, respectively (**Figures 1G–L**). Integration across all methods converged on a four-gene signature comprising *LAIR1*, *PLAC8*, *ACSS2*, and *PCOLCE2* (**Figure 1M**), which was carried forward for independent cohort validation.

### 3.2 Development and cross-cohort validation of the four-gene diagnostic model

Expression analysis showed that *LAIR1*, *PLAC8*, *ACSS2*, and *PCOLCE2* were significantly upregulated in patients with postoperative sepsis in GSE131761 (**Figure 2A**). Consistent expression patterns were observed in GSE74224, which compared critically ill patients with sepsis with patients with postoperative infection-negative systemic inflammation (**Figure 2B**). Upregulation of all four genes was also confirmed in E-MTAB-1548, an independent surgical cohort comprising patients with sepsis and those with non-infectious SIRS (**Figure 2C**). Using GSE131761 as the model-development cohort, a four-gene diagnostic model was constructed and visualized as a nomogram (**Figure 2D**). The model demonstrated strong calibration between predicted and observed risk (**Figure 2E**) and outperformed individual genes in decision curve analysis across a broad range of threshold probabilities, indicating improved net clinical benefit (**Figure 2F**). Clinical impact analysis further supported robust discrimination of high-risk cases (**Figure 2G**; **Supplementary Figure 3**). ROC analysis showed that the four-gene model achieved an area under the curve (AUC) of 0.865 in the GSE131761 development cohort, 0.965 in GSE74224, and 0.827 in the independent E-MTAB-1548 surgical validation cohort (**Figures 2H–J**; **Table 1**).

**Figure 2:**
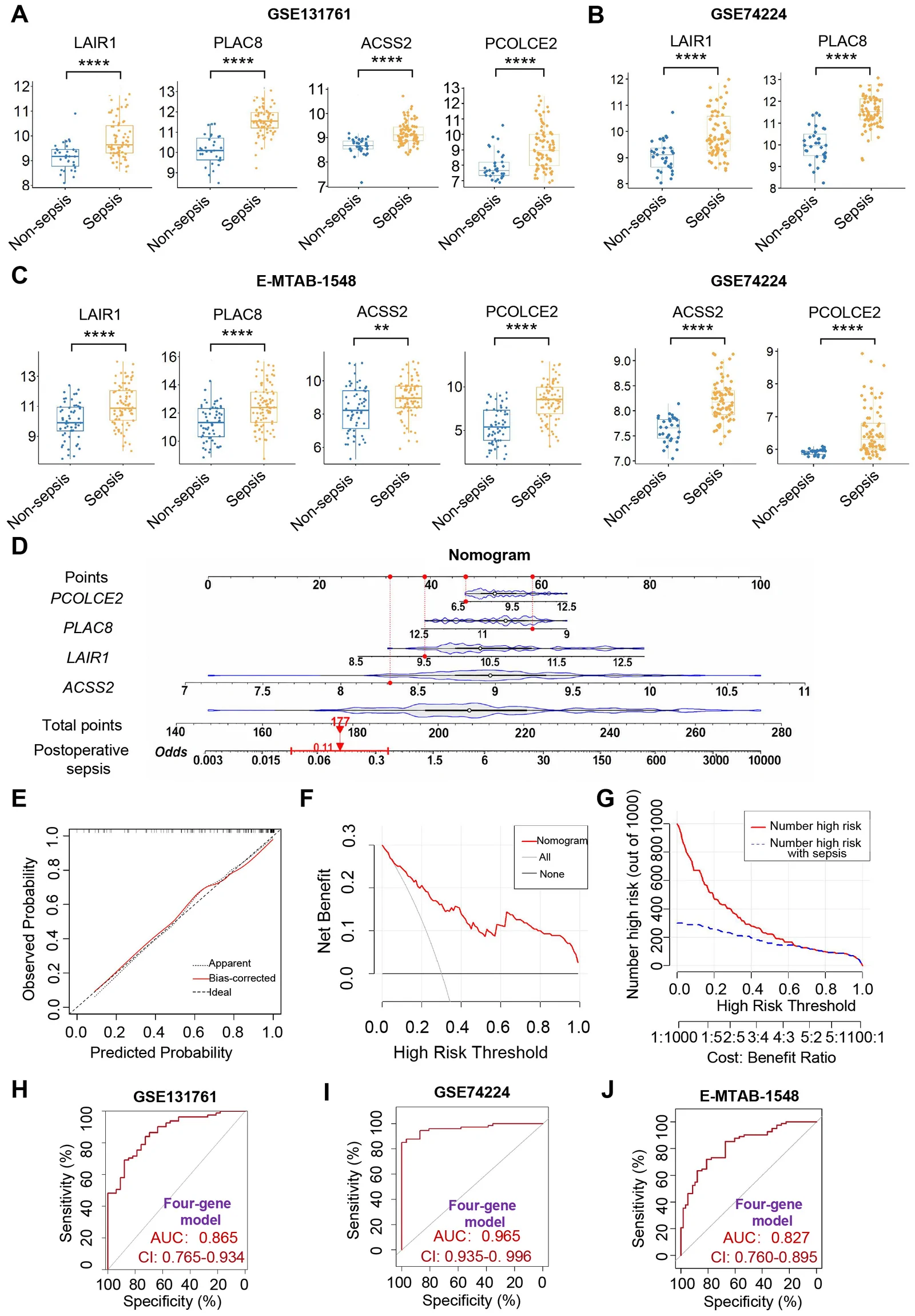
Development and cross-cohort validation of the four-gene diagnostic model. **(A, B)** Validation of hub genes (*LAIR1, PLAC8, ACSS2*, and *PCOLCE2*) in GSE131761 **(A)** and GSE74224 **(B)** datasets. **(C)** External validation of four hub genes in E-MTAB-1548 dataset. **(D)** Development of a diagnostic nomogram based on the four hub genes using the GSE131761 training cohort to predict the risk of postoperative sepsis. **(E–G)** Evaluation of the nomogram’s clinical utility. The calibration curve **(E)** illustrates the agreement between predicted and observed probabilities. DCA **(F)** shows the net benefit of the nomogram across various threshold probabilities. The CIC **(G)** displays the estimated number of high-risk cases versus the actual number of cases with sepsis. **(H–J)** ROC curves assessing the discriminative performance of the four-gene model in GSE131761 **(H)**, GSE74224 **(I)**, and E-MTAB-1548 **(J)**. AUC values and 95% confidence intervals **(CI)** are indicated. Data are shown as box-and-whisker plots **(A–C)**. Statistical significance was assessed using the student’s t test **(A–C)**. **p < 0.01, ****p < 0.0001. DCA decision curve analysis, CIC clinical impact curve, ROC receiver operating characteristic.

**Table 1:** Diagnostic performance across the discovery and validation cohorts.

Cohort	Gene	AUC	95% CI
GSE131761	LAIR1	0.802	0.722–0.883
	PLAC8	0.704	0.608–0.800
	ACSS2	0.842	0.770–0.913
	PCOLCE2	0.767	0.673–0.862
	Four-gene model	0.865	0.765–0.934
GSE74224	LAIR1	0.788	0.697–0.879
	PLAC8	0.922	0.868–0.977
	ACSS2	0.896	0.838–0.955
	PCOLCE2	0.843	0.768–0.917
	Four-gene model	0.965	0.935–0.996
E-MTAB-1548	LAIR1	0.706	0.620–0.791
	PLAC8	0.719	0.635–0.823
	ACSS2	0.646	0.550–0.742
	PCOLCE2	0.814	0.775–0.884
	Four-gene model	0.827	0.760–0.895
GSE65682	LAIR1	0.824	0.738-0.919
	PLAC8	0.912	0.854-0.969
	ACSS2	0.737	0.635-0.839
	PCOLCE2	0.917	0.852-0.982
	Four-gene model	0.929	0.873-0.985
GSE77167	LAIR1	0.898	0.810-0.985
	PLAC8	0.939	0.878-0.998
	ACSS2	0.878	0.789-0.968
	PCOLCE2	0.989	0.967-1.000
	Four-gene model	0.997	0.990-1.000

We next evaluated the model in a clinically defined subset of GSE65682. This cohort included 51 patients with abdominal-source sepsis and 42 postoperative controls who underwent gastrointestinal surgery for non-infectious conditions. All four genes were significantly upregulated in abdominal sepsis (**Supplementary Figure 4A**) and showed variable individual discriminative performance (**Supplementary Figure 4B**). The combined four-gene model achieved an AUC of 0.929 (95% CI, 0.873–0.985), supporting its reproducibility in distinguishing abdominal sepsis from non-infectious postoperative inflammation (**Supplementary Figure 4C**; **Table 1**). We then assessed the reproducibility of the model in an independent postoperative cohort using GSE77167, which included 32 patients who developed intra-abdominal infection after colorectal cancer surgery and 30 patients with an uncomplicated postoperative course. All four genes were significantly upregulated in the postoperative infection group (**Supplementary Figure 4D**). LAIR1, PLAC8, ACSS2, and PCOLCE2 individually demonstrated strong diagnostic discrimination, with AUCs of 0.898 (95% CI, 0.810–0.985), 0.939 (95% CI, 0.878–0.998), 0.878 (95% CI, 0.789–0.968), and 0.989 (95% CI, 0.967–1.000), respectively (**Supplementary Figure 4E**). The combined four-gene model achieved an AUC of 0.997 (95% CI, 0.990–1.000), representing the highest AUC among the evaluated predictors (**Supplementary Figure 4F**; **Table 1**).

Collectively, these findings demonstrate the reproducibility of the four-gene model and support its potential for distinguishing infection-associated sepsis from infection-negative systemic inflammation in both surgical and broader sepsis populations.

### 3.3 Characterization of a murine postoperative endotoxemia model

To validate the *in vivo* relevance of the identified hub genes and enable subsequent mechanistic investigation, a postoperative endotoxemia model was established by combining surgical injury with subsequent LPS challenge, whereas sham and surgery-only mice served as controls. Surgery-plus-LPS mice exhibited markedly increased mortality, with significantly reduced 7-day survival compared with both control groups (**Figure 3A**). Consistent with severe systemic inflammation, serum IL-6, IL-1β, and TNF-α levels were markedly elevated at 24 h in surgery-plus-LPS mice (**Figure 3B**).

**Figure 3:**
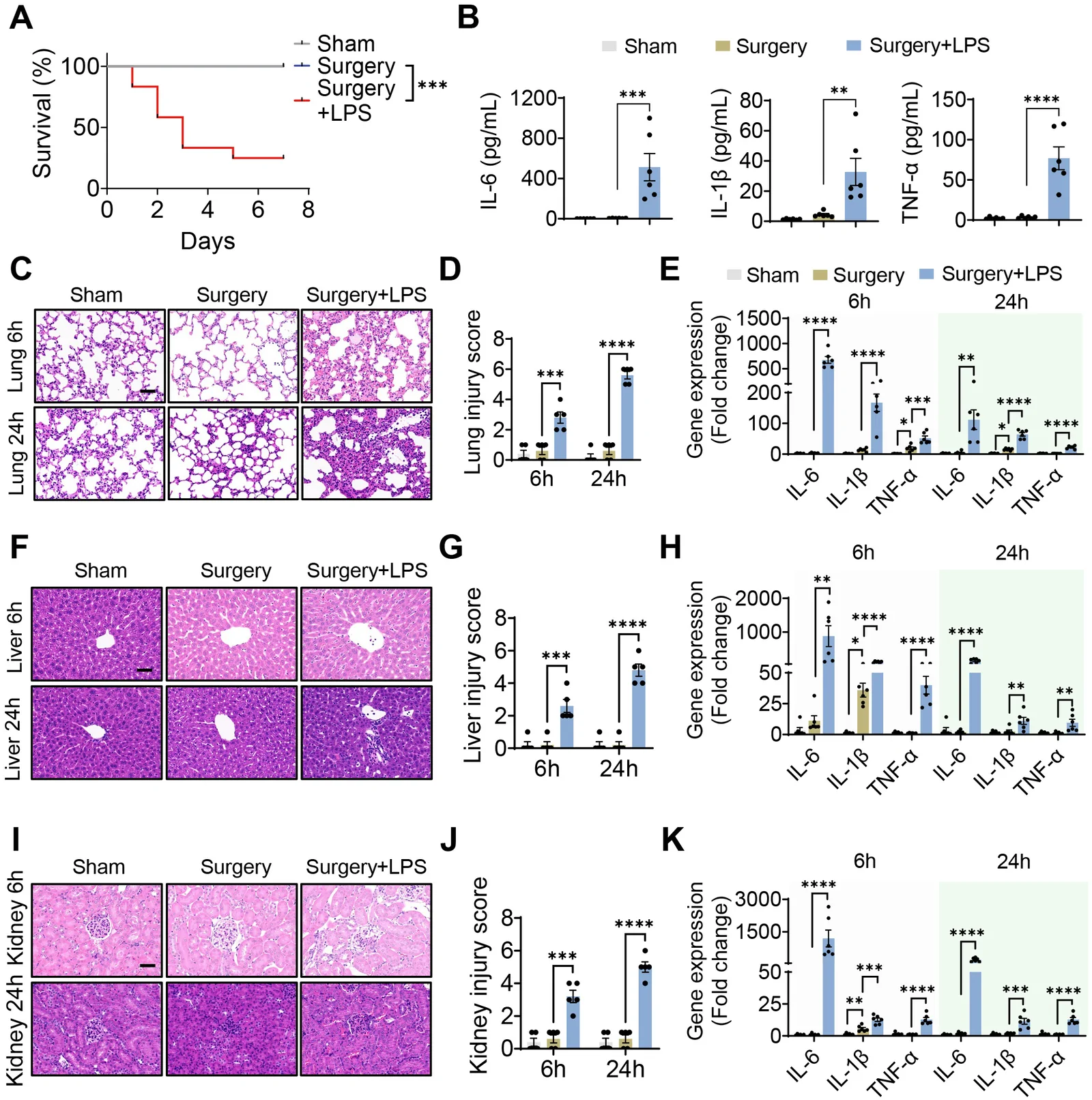
Characterization of a murine postoperative endotoxemia model. **(A)** Kaplan–Meier survival analysis of mice in the Sham, Surgery, and Surgery+LPS groups (n = 12 per group). **(B)** Serum concentrations of IL-6, IL-1β, and TNF-α quantified by ELISA at 24 h post-modeling (n = 6 per group). **(C–E)** Representative H&E images **(C)**, pathological scores [**(D)**, n = 5 per group], and relative mRNA expression of inflammatory cytokines (*IL-6*, *IL-1β*, and *TNF*-*α*) [**(E)**, n = 6 per group] in lung tissues at 6 h and 24 h. Scale bars = 50 μm. **(F–H)** Representative H&E images **(F)**, pathological scores [**(G)**, n = 5 per group], and relative mRNA levels of cytokines [**(H)**, n = 6 per group] in liver tissues at 6 h and 24 h. Scale bars = 50 μm. **(I–K)** Representative H&E images **(I)**, pathological scores [**(J)**, n = 5 per group], and relative mRNA levels of cytokines [**(K)**, n = 6 per group] in kidney tissues at 6 h and 24 h. Scale bars = 50 μm. Data are presented as mean ± SEM; each dot represents an individual mouse. Statistical significance was determined by log-rank (Mantel–Cox) test for survival **(A)** and one-way ANOVA followed by Tukey’s *post hoc* test for multiple comparisons **(B, D, E, G, H, J, K)**. *p < 0.05 **p < 0.01, ***p < 0.001, ****p < 0.0001. H&E hematoxylin and eosin.

Histological analysis revealed progressive multiorgan injury involving the lung, liver, and kidney. Mild inflammatory changes were detectable at 6 h, whereas severe tissue injury became evident by 24 h, accompanied by significantly increased pathological scores and sustained local cytokine upregulation across all organs (**Figures 3C–K**). In contrast, surgery alone induced only mild and organ-restricted inflammatory responses without progression to systemic tissue injury. Collectively, these findings show that LPS challenge in the context of preceding surgical injury induces severe systemic inflammation, progressive multiorgan damage, and increased mortality.

### 3.4 Dynamic expression profiling reveals distinct patterns among the four candidate genes

To determine whether the identified hub genes were dynamically regulated during *in vivo* progression of postoperative sepsis, we profiled the expression of *Lair1*, *Plac8*, *Acss2*, and *Pcolce2* in the peripheral blood and major organs at 6 h and 24 h post-model induction (**Figure 4A**). In PBMCs, the expression patterns of the four genes differed over time (**Figure 4B**). Lair1 and Plac8 were markedly induced in the Surgery+LPS group at 6 h. Lair1 expression increased further at 24 h, whereas Plac8 expression was significantly lower at 24 h than at 6 h, indicating distinct temporal profiles in circulating immune cells. In contrast, Acss2 and Pcolce2 showed less pronounced changes in PBMCs.

**Figure 4:**
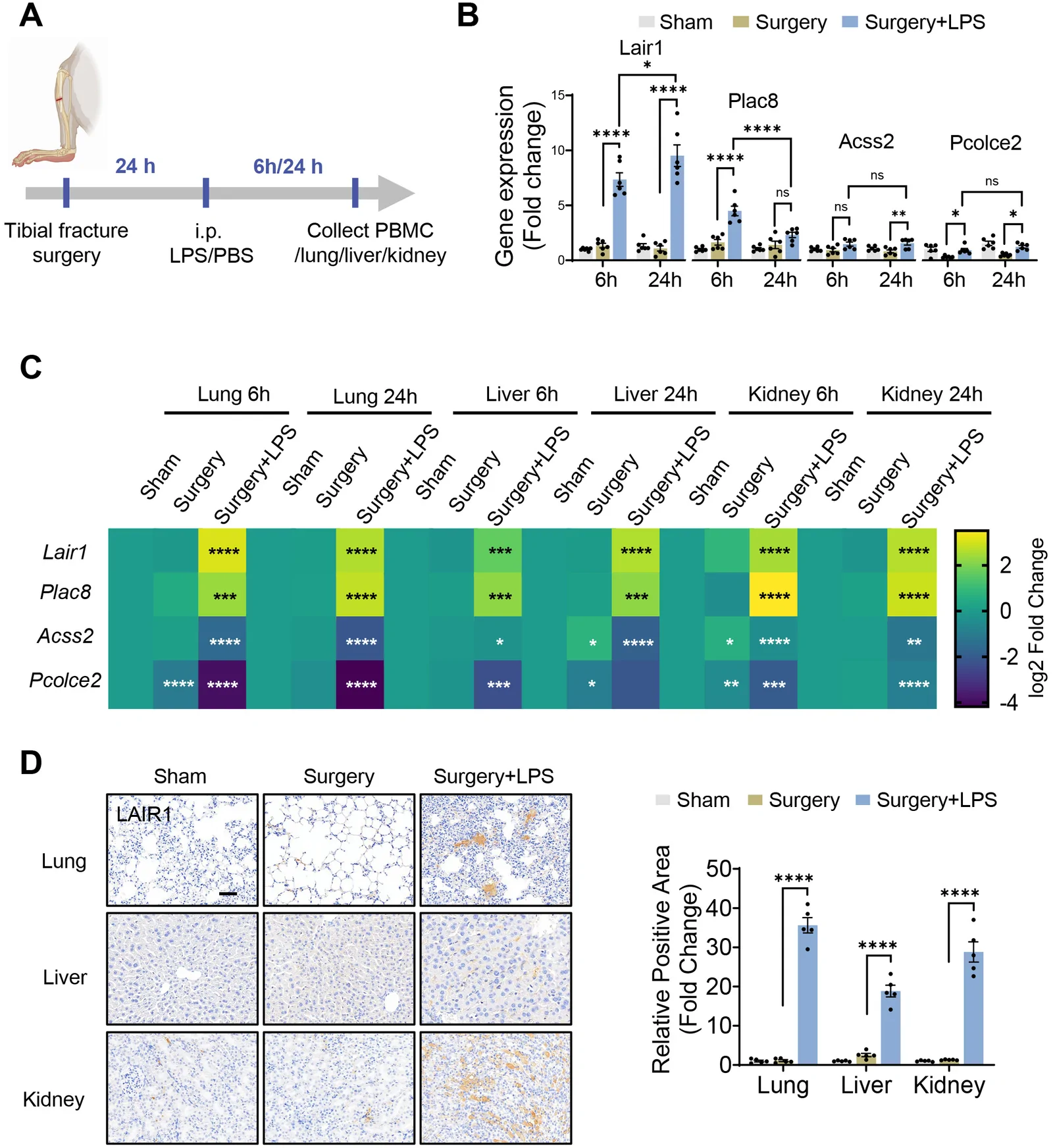
Dynamic expression profiling reveals distinct patterns among the four candidate genes. **(A)** Schematic timeline illustrating the experimental design of the postoperative endotoxemia model and sample collection. **(B)** Validation of candidate hub gene expression *(Lair1, Plac8, Acss2*, and *Pcolce2*) in PBMCs at 6 h and 24 h post-modeling, as determined by RT‐qPCR (n = 6 per group). **(C)** Heatmap summarizing the relative mRNA expression levels of candidate genes across the lung, liver, and kidney at 6 h and 24 h. Data are presented as *log*_2_ fold changes relative to the Sham group. (n = 6 per group). **(D)** Representative immunohistochemistry images (left) and corresponding quantification (right) of LAIR1 protein expression in lung, liver, and kidney tissues at 24 h (n = 5 per group). Scale bars = 50 μm. Data are presented as mean ± SEM; each dot represents an individual mouse. Statistical significance was determined by two-way ANOVA, followed by Tukey’s multiple-comparisons test **(B)**, and one-way ANOVA followed by Tukey’s *post hoc* test **(C, D)**. In the heatmap **(C)**, asterisks in the Surgery group indicate comparisons vs. Sham; asterisks in the Surgery+LPS group indicate comparisons vs. Surgery. *p < 0.05, **p < 0.01, ***p < 0.001, ****p < 0.0001; ns, not significant. PBMCs peripheral blood mononuclear cells.

The organ data showed clear compartment-specific differences among the four genes. Both Lair1 and Plac8 were significantly upregulated in the lung, liver, and kidney at 6 and 24 h after surgery plus LPS challenge (**Figure 4C**; **Supplementary Figure 5**). In contrast, Acss2 and Pcolce2 were generally reduced in these peripheral organs. Consistently, LAIR1 and PLAC8 protein levels were significantly increased in the Surgery+LPS group compared with the corresponding Surgery group at both 6 and 24 h (**Supplementary Figure 6**). LAIR1 expression showed a further increase between 6 and 24 h, whereas PLAC8 levels remained relatively stable over time. In contrast, ACSS2 and PCOLCE2 protein levels were reduced after LPS challenge at both time points. Notably, Plac8 displayed sustained induction in peripheral organs despite its decline in PBMCs at 24 h, suggesting that its regulation differs between circulating and tissue compartments. LAIR1 was selected for subsequent mechanistic investigation based on its sustained and multi-organ induction, although PLAC8 remained a biologically relevant candidate for future functional studies.

Immunohistochemical staining further confirmed marked accumulation of LAIR1 protein in the lung, liver, and kidney at 24 h (**Figure 4D**), providing additional tissue-level evidence for its sustained upregulation during postoperative endotoxemia.

### 3.5 Single-cell analysis reveals monocyte-enriched LAIR1 expression and altered immune signaling in sepsis

To define the cellular context of LAIR1, scRNA-seq data from PBMCs (GSE167363) were analyzed, identifying 10 immune cell populations (**Figure 5A**). Sepsis was associated with expansion of the monocyte compartment (**Figure 5B**). LAIR1 expression was highly enriched in monocytes and minimally detected in other immune subsets, indicating a monocyte-enriched expression pattern (**Figures 5C, D**). To validate this cellular localization at the tissue level, we performed dual immunofluorescence staining for LAIR1 and the macrophage marker F4/80 in mouse lung tissue. Extensive colocalization of LAIR1 with F4/80-positive cells was observed in the surgery+LPS group (**Figures 5E, F**). This finding provided tissue-level confirmation of the monocyte-enriched expression pattern identified by the single-cell analysis. Within the single-cell dataset, monocyte LAIR1 expression was significantly higher in both survivors and non-survivors with sepsis than in healthy controls (**Figure 5G**).

**Figure 5:**
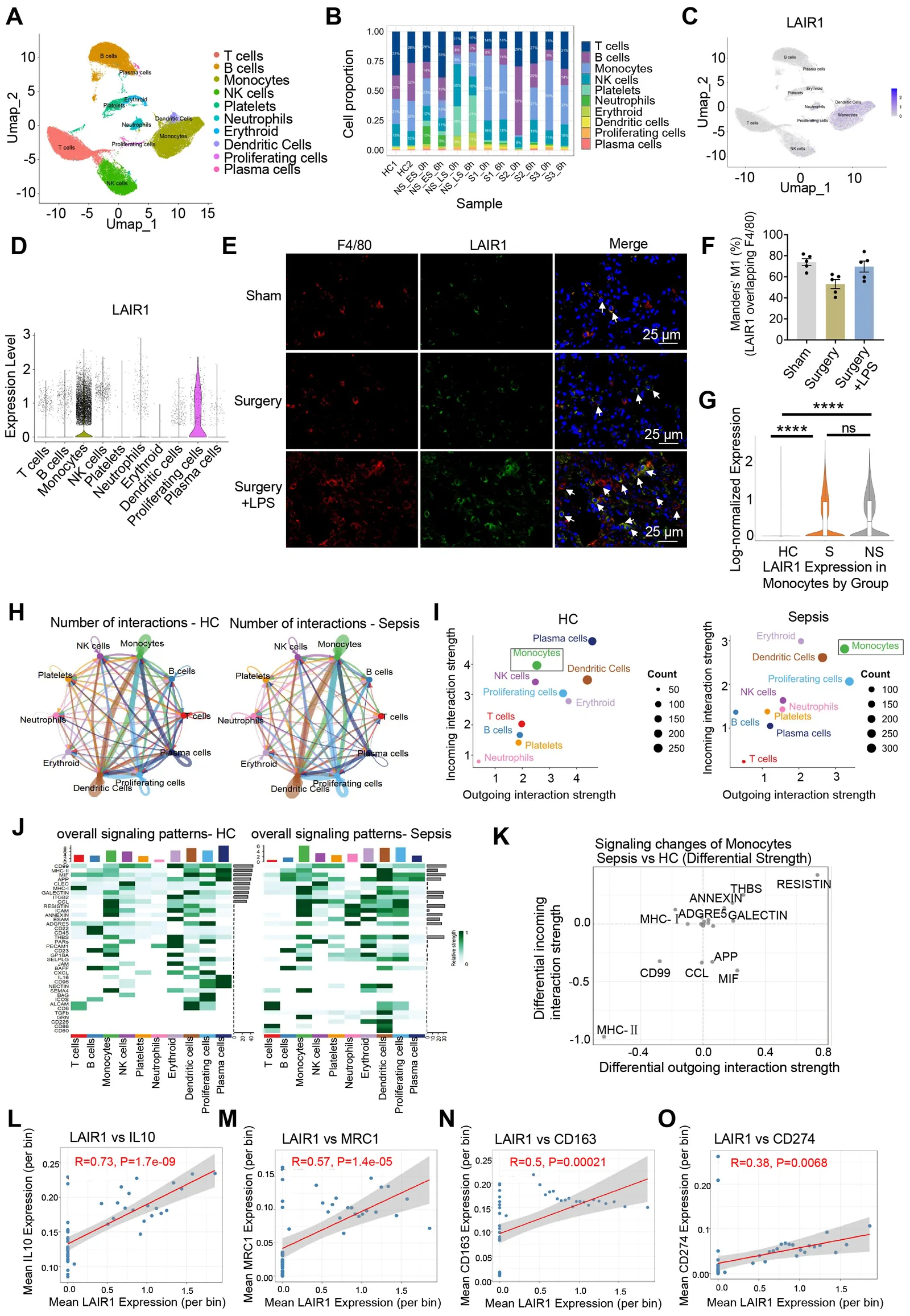
Single-cell analysis reveals monocyte-enriched LAIR1 expression and altered immune signaling in sepsis. **(A)** UMAP visualization of the transcriptional landscape of human PBMCs from the public dataset GSE167363, with cell clusters annotated and color-coded. **(B)** Bar plots illustrating the relative proportions of major immune cell populations across individual PBMC samples. **(C)** Feature plot showing the cell-type-specific expression profile of LAIR1 mRNA across the UMAP projection. **(D)** Violin plot depicting LAIR1 expression levels across various immune cell types. **(E)** Representative dual-immunofluorescence images of F4/80 (red), LAIR1 (green), and DAPI (blue) in mouse lung tissues. Arrowheads indicate representative regions of spatial overlap between LAIR1 and F4/80 signals. Scale bars = 25 μm. **(F)** Quantification of LAIR1 colocalization with F4/80-positive cells was performed using Manders’ M1 coefficient, representing the fraction of LAIR1 fluorescence overlapping with F4/80 fluorescence. (n = 5 mice per group). Each dot represents one mouse; data are presented as mean ± SEM. **(G)** Comparative analysis of LAIR1 expression in monocytes across clinical cohorts: healthy controls (HC), non-surviving sepsis patients (NS), and surviving sepsis patients (S). **(H)** Circle plots illustrating the total number of inferred cell-to-cell interactions in the HC and Sepsis groups, analyzed using the “CellChat” R package. **(I)** Scatter plots depicting the outgoing and incoming interaction strengths of various cell populations in HC and Sepsis cohorts. **(J)** Heatmap showing the relative strength of overall signaling patterns across cell types in the two groups. **(K)** Scatter plot showing differential incoming and outgoing signaling strengths in monocytes between Sepsis and HC. **(L–O)** Binned correlation analysis between LAIR1 and IL10 **(L)**, MRC1 **(M)**, CD163 **(N)**, and CD274 **(O)**. Statistical significance was determined by Wilcoxon rank-sum test **(G)** and Spearman’s rank correlation analysis **(L–O)**. ****p < 0.0001; ns, not significant. HC healthy control, NS_ES non-survived septic patient, early-stage, NS_LS non-survived septic patient, late-stage, S survivor sepsis patients.

Cell–cell communication analysis identified monocytes as central signaling hubs in sepsis, exhibiting increased interaction density, particularly with dendritic cells (**Figure 5H**). Compared with healthy controls, septic monocytes showed enhanced outgoing signaling strength but reduced incoming signaling (**Figure 5I**), indicating a shift in communication directionality.

Pathway-level analysis demonstrated concurrent activation of inflammatory signaling programs (e.g., RESISTIN, THBS) and immunoregulatory pathways, including GALECTIN and ANNEXIN signaling, together with suppression of MHC-II–associated communication (**Figures 5J, K**). This pattern indicates a coordinated shift toward mixed inflammatory–immunoregulatory monocyte states. Consistently, LAIR1 expression positively correlated with immunoregulatory markers including IL10, MRC1, CD163, and CD274 (**Figures 5L–O**), supporting its association with regulatory monocyte phenotypes during sepsis.

### 3.6 LAIR1 blockade exacerbates systemic inflammation and multiorgan injury in postoperative endotoxemia model

To determine the functional role of LAIR1 in surgery+LPS model, mice were treated with a neutralizing anti-LAIR1 antibody (αLAIR1) or isotype control in the surgery+LPS model (**Figure 6A**). LAIR1 blockade significantly reduced 7-day survival compared with IgG-treated controls (**Figure 6B**). This was accompanied by a marked increase in systemic inflammatory cytokines, including IL-6, IL-1β, and TNF-α (**Figures 6C–E**), indicating amplification of the systemic inflammatory response upon LAIR1 inhibition. Histological analysis revealed that αLAIR1 treatment markedly aggravated multiorgan injury in surgery+LPS mice. Severe pathological deterioration was observed in the lung, liver, and kidney, characterized by exacerbated inflammatory infiltration, tissue structural disruption, and significantly increased injury scores across all organs (**Figures 6F, G**). Collectively, LAIR1 neutralization aggravated mortality and systemic inflammatory injury, indicating that LAIR1 acts as a negative regulator of inflammation during postoperative endotoxemic response.

**Figure 6:**
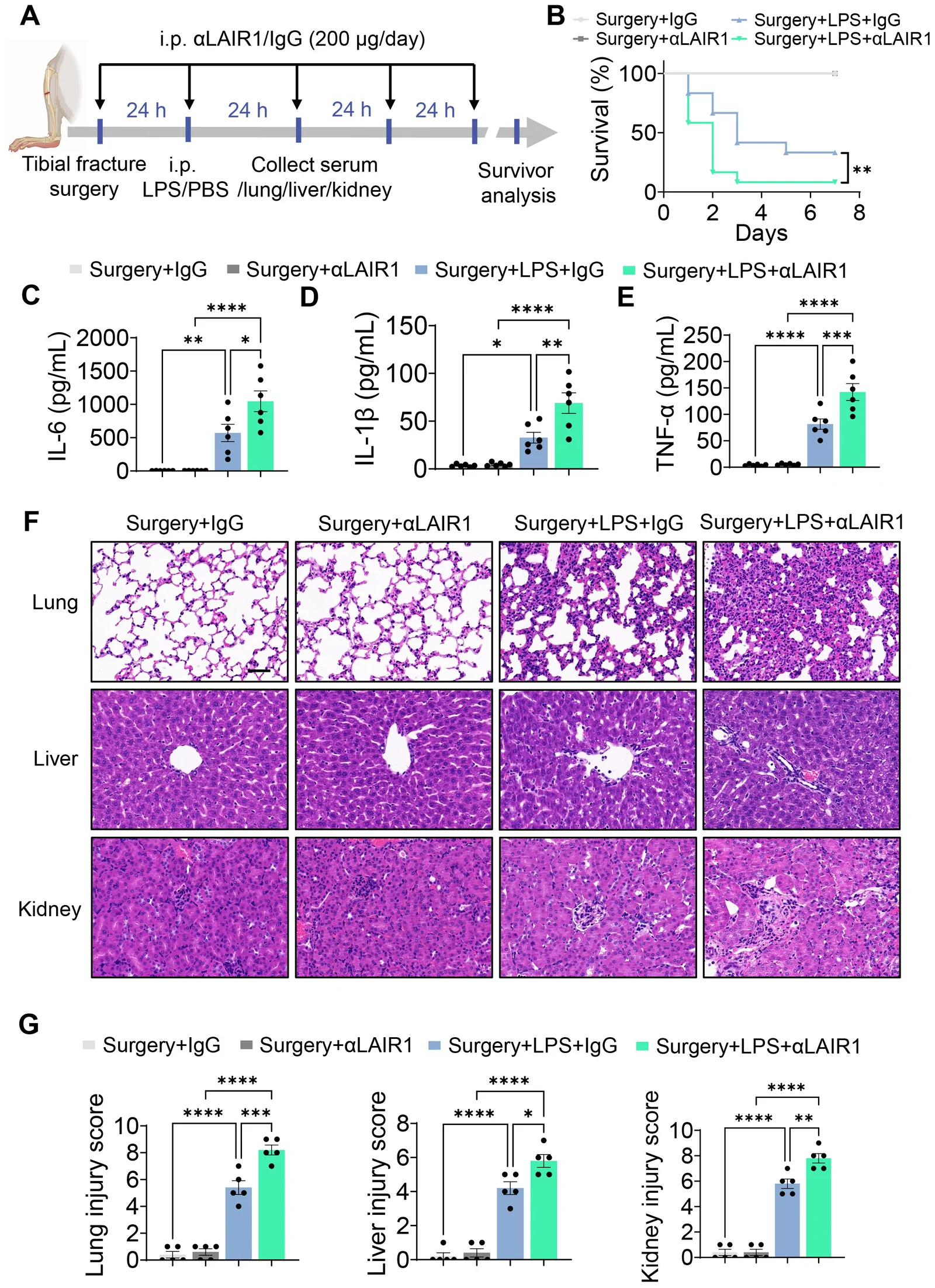
LAIR1 blockade exacerbates systemic inflammation and multiorgan injury in postoperative endotoxemia model. **(A)** Schematic illustration of the experimental timeline and the *in vivo* administration protocol for αLAIR1 neutralizing antibody. **(B)** Kaplan–Meier survival curves for mice in the indicated groups: Surgery+IgG, Surgery+αLAIR1, Surgery+LPS+IgG, and Surgery+LPS+αLAIR1 (n = 12 per group). **(C–E)** Serum levels of IL-6 **(C)**, IL-1β **(D)**, and TNF-α **(E)** quantified by ELISA at 24 h after LPS or PBS administration (n = 6 per group). **(F, G)** Representative H&E images **(F)** and corresponding histopathological scores **(G)** of lung, liver, and kidney tissues at 24 h after LPS or PBS administration (n = 5 per group). Scale bars = 50 μm. Data are presented as mean ± SEM; each dot represents one individual mouse. Survival analysis was performed using the log‐rank (Mantel–Cox) test **(B)**. Multiple-group comparisons were analyzed by one‐way ANOVA followed by Tukey’s *post-hoc* test **(C, D, E, G)**. *p < 0.05, **p < 0.01, ***p < 0.001, ****p < 0.0001. H&E hematoxylin and eosin.

## 4 Discussion

Postoperative sepsis remains a major challenge in perioperative medicine due to the complex interaction between surgery-induced sterile inflammation and subsequent infectious insult, resulting in dysregulated immune responses and high mortality. In this study, we integrated DEGs analysis, WGCNA, and machine-learning approaches to identify a four-gene diagnostic signature (LAIR1, PLAC8, ACSS2, and PCOLCE2), which was validated across discovery and independent cohorts. Multi-level validation using bulk transcriptomics, single-cell RNA sequencing, and *in vivo* functional validation consistently highlighted LAIR1 as a monocyte-associated immunoregulatory molecule involved in perioperative immune adaptation during sepsis.

Early discrimination of postoperative sepsis from sterile postoperative inflammation remains clinically challenging because surgical injury alone induces profound systemic immune activation that can mimic early infection (36). Unlike conventional sepsis biomarkers that primarily distinguish infection from healthy states (12), biomarkers intended for postoperative use must distinguish infection-associated immune dysregulation from an existing background of surgery-induced inflammation. To address this challenge, we developed a four-gene model presented as a nomogram and evaluated its performance in three independent surgical cohorts. Consistent diagnostic performance was observed in E-MTAB-1548, GSE65682, and GSE77167, which included postoperative sepsis, surgically managed abdominal sepsis, and postoperative intra-abdominal infection, respectively. The concordant findings across these independent cohorts support the reproducibility of the signature and its ability to capture infection-associated immune dysregulation in the postoperative setting. Prospective multicenter studies will be needed to further establish its clinical applicability.

In the postoperative endotoxemia model, the four-gene signature exhibited distinct temporal and spatial expression patterns across circulating immune cells and peripheral organs. In PBMCs, all four genes were induced following combined surgical injury and LPS challenge. PLAC8 showed a moderate increase without clear temporal amplification, consistent with its reported role in promoting monocyte activation and ERK-dependent inflammatory signaling, indicating a more transient association with early immune activation rather than sustained regulation (37). By comparison, ACSS2 and PCOLCE2 displayed a distinct trajectory in peripheral organs relative to circulating immune cells, with reduced expression in lung, liver, and kidney at later stages. Such compartment-specific regulation suggests that circulating immune activation and organ-level dysfunction are partially uncoupled during disease progression (38). In contrast, LAIR1 showed progressive and sustained upregulation from 6 h to 24 h, with sustained induction in PBMCs and consistent upregulation across multiple organs, suggesting a persistent response across the circulation–tissue axis.

Perioperative immune dysregulation is initiated by tissue injury, release of damage-associated signals followed by a compensatory immunoregulatory response. LAIR1 may function as an important checkpoint within this transition. It is broadly expressed by myeloid cells and enriched in circulating monocytes and tissue macrophages. In our study, single-cell RNA sequencing showed monocyte-enriched LAIR1 expression, whereas dual immunofluorescence localized LAIR1 to F4/80-positive macrophages. LAIR1 contains immunoreceptor tyrosine-based inhibitory motifs (ITIMs) and can recruit SH2 domain of phosphatases 1/2 (SHP-1/2), thereby attenuating inflammatory signaling (39, 40). Consistent with this function, LAIR1 activation suppresses proinflammatory M1-like macrophage differentiation and promotes an alternative phenotype (41). In our study, LAIR1 expression was positively associated with IL10, MRC1, CD163, and CD274, indicating that LAIR1 induction accompanies an immunoregulatory transcriptional program. Increased LAIR1 expression may participate in the reprogramming of monocytes from a predominantly proinflammatory state toward a more immunoregulatory phenotype. The exacerbated inflammation and organ injury following LAIR1 blockade are consistent with this potential role, although direct causality and the cell-specific contribution of monocyte LAIR1 remain to be established.

Cell–cell communication analysis demonstrated that monocytes undergo extensive remodeling of intercellular signaling networks during sepsis. Monocytes displayed increased connectivity and enhanced signaling activity, indicating a dynamically activated immune state with concurrent regulatory adaptation. Against this background of immune activation, LAIR1 upregulation may represent a compensatory negative-feedback response that restrains excessive inflammation. Supporting this interpretation, systemic LAIR1 blockade in the postoperative endotoxemia model reduced survival and increased cytokine release and multiorgan injury. These findings suggest that endogenous LAIR1 signaling helps maintain immune balance when surgical injury is followed by an endotoxin challenge. Increased LAIR1 expression may therefore reflect an adaptive response to inflammatory stress during postoperative immune dysregulation.

Several limitations should be acknowledged. First, the transcriptomic datasets analyzed were retrospective and lacked detailed perioperative clinical parameters, limiting direct clinical correlation. Second, the surgery+LPS model reflects acute endotoxemic inflammation but does not fully recapitulate clinical postoperative sepsis. Further validation in infection-based models, such as cecal ligation and puncture (CLP), is therefore required. Third, mechanistic interpretation of LAIR1 is limited by transcriptomic association and systemic antibody blockade. Because anti-LAIR1 treatment may affect multiple LAIR1-expressing immune-cell populations, the observed effects cannot be attributed specifically to monocyte LAIR1. Future studies using myeloid- or monocyte-specific genetic models are needed to establish cell-type-specific causality.

## 5 Conclusion

The four-gene signature reproducibly distinguished sepsis from infection-negative systemic inflammation across surgical and broader ICU cohorts. Blockade of LAIR1 exacerbated systemic inflammation, multiorgan injury, and mortality in postoperative endotoxemia. These findings support the diagnostic potential of the four-gene signature and a protective immunoregulatory role for LAIR1 in postoperative sepsis.

## Data Availability

The original contributions presented in the study are included in the article/**Supplementary Material**. Further inquiries can be directed to the corresponding author.
