# Supplementary material for: Integrated transcriptomic and functional analyses identify LAIR1 as a key immunoregulatory molecule in postoperative sepsis
Source: Front Immunol. 2026 Oct 14;17:1931628. doi: 10.3389/fimmu.2026.1931628 (PMC13713299; doi:10.3389/fimmu.2026.1931628)
Supplement: Supplementary file 1 [file DataSheet1.docx]

**Supplementary Figure** **1**

**Supplementary Figure 1. Gene functional enrichment analysis using Metascape.** (A) Metascape enrichment analysis of the DEGs. (B) The top-level biological process identified in DEGs. (C) Network of enriched terms using the Metascape tool. DEGs, differentially expressed genes

**Supplementary Figure 2**

**Supplementary Figure 2.** **WGCNA and functional enrichment analysis of brown-module genes.** **(A)** Sample clustering was conducted to detect and remove outliers. **(B)** Scale-free fit and mean connectivity for different soft-thresholding powers. **(C)** Gene clustering dendrogram generated by WGCNA. **(D)** Metascape enrichment analysis of genes in the brown module. **(E)** Network of enriched terms for brown-module genes, generated using Metascape.

**Supplementary Figure 3**

**Supplementary Figure 3. Clinical impact curves for the individual diagnostic genes in GSE131761. (A–D)** Clinical impact curves for LAIR1, PLAC8, ACSS2, and PCOLCE2 respectively. The red curve indicates the number of individuals classified as high risk at each threshold probability, whereas the blue curve indicates the number of high-risk individuals with the outcome.

**Supplementary Figure 4**

**Supplementary Figure 4. Evaluation of the four-gene diagnostic signature in GSE65682 and GSE77167. (A–C)** Expression levels of *LAIR1*, *PLAC8*, *ACSS2*, and *PCOLCE2* (A), ROC curves for individual genes (B), and the four-gene model (C) in GSE65682. **(D–F)** Expression levels of LAIR1, PLAC8, ACSS2, and PCOLCE2 (D), ROC curves for individual genes (E), and the four-gene model (F) in GSE77167. AUCs and 95% confidence intervals are shown. Statistical significance was assessed using the student’s t test **(A, D)**. ****P < 0.0001. ROC, receiver operating characteristic; AUC, area under the curve; CI, confidence interval.

**Supplementary Figure 5**

**Supplementary Figure 5. Individual-level expression of the four candidate genes in the postoperative endotoxemia model. (A–C)** Individual expression levels of *Lair1*, *Plac8*, *Acss2*, and *Pcolce2*, measured by quantitative RT-qPCR in the lung (A), liver (B), and kidney (C) at 6 and 24 h after LPS or PBS administration. Each point represents an individual animal. Data are presented as mean ± SEM (n = 6 per group). Statistical significance was determined by one-way ANOVA followed by Tukey’s post hoc test **(A-C)**.*P < 0.05, **P < 0.01, ***P < 0.001, and ****P < 0.0001.

**Supplementary Figure 6**

**Supplementary Figure 6. Protein expression of the four candidate genes in lung tissue during postoperative endotoxemia.** Representative western blots and quantification of the relative protein expression levels of LAIR1, PLAC8, ACSS2, and PCOLCE2 in lung tissue from Surgery and Surgery+LPS mice at 6 and 24 h after LPS or PBS administration. Protein expression was normalized to GAPDH. Data are presented as mean ± SEM (n = 3 per group). Statistical significance was assessed by one-way ANOVA followed by Tukey’s multiple-comparisons test. *P < 0.05, **P < 0.01, ****P < 0.0001; ns, not significant.

**Supplementary Table 1. Dataset characteristics and clinical comparisons**

| Dataset | Platform | Case group, n | Comparator group, n |
| --- | --- | --- | --- |
| GSE131761 | GPL13497 | Postoperative septic shock, 81 | Postoperative non-septic shock, 33 |
| GSE74224 | GPL5175 | Sepsis, 74 | Postoperative infection-negative systemic inflammation, 31 |
| E-MTAB-1548 | A-MEXP-2183 | Postoperative sepsis, 82 | Postoperative  non-infectious SIRS, 58 |
| GSE65682 subset | GPL13667 | Abdominal sepsis, 51 | Postoperative  GI surgery controls, 42 |
| GSE77167 | GPL6244 | Postoperative intra-abdominal infection, 32 | Uncomplicated postoperative recovery, 30 |

Abbreviations: GI, gastrointestinal; SIRS, systemic inflammatory response syndrome.

**Supplementary Table 2. Sampling context, sample selection, and analytical roles**

| Dataset | Blood sampling time | Samples included | Role in this study |
| --- | --- | --- | --- |
| GSE131761 | Within 24 h of clinical shock diagnosis | 114; healthy controls excluded | Primary discovery and model development |
| GSE74224 | Cases: within 24 h of ICU admission; controls: within 24 h of surgery | 105 | Complementary discovery |
| E-MTAB-1548 | Within 24 h of sepsis or SIRS diagnosis in the associated study subset | 140; healthy controls excluded | Postsurgical four-gene evaluation |
| GSE65682 subset | Within 24 h of ICU admission in the associated abdominal study | 93; selected abdominal-sepsis/GI-control subset | Complementary evaluation in abdominal sepsis |
| GSE77167 | Postoperative sampling | 62; matched and unmatched samples | Complementary evaluation in postoperative infection |

**Supplementary Table 3. Primer sequences used in quantitative PCR**

| Gene | Sense (5’-3’) | Antisense (5’-3’) |
| --- | --- | --- |
| Gapdh | TGGTCCTCAGTGCCCAAG | CTGCCCAGAACATCATCCCT |
| Il6 | CTGCAAGAGACTTCCATCCAG | AGTGGTATAGACAGGTCTGTTGG |
| Il1b | TGGATGCTCTCATCAGGACAG | GAAATGCCACCTTTTGACAGTG |
| Tnf-α | AGGCGGTGCCTATGTCTCAG | GCTCCTCCACTTGGTGGTTT |
| Lair1 | GACCATCACTCCCTCACACA | GGACCTAGCTTTCCACACCAG |
| Plac8 | CAGGAGGAGAGCCATGAACG | CTGAAGGAGTGTGTGCTGAGT |
| Acss2 | GGCACAGTAGGTGAACCCAT | AGGCGCTACACCGAAGAATG |
| Pcolce2 | GTCAGCAGAAGTGTAGACGGAT | CCAGGTTGCCTTCCCTGTAG |
